# Differences in gene expression profile between vocal cord Leukoplakia and normal larynx mucosa by gene chip

**DOI:** 10.1186/s40463-018-0260-4

**Published:** 2018-02-12

**Authors:** Jianhua Peng, He Li, Jun Chen, Xianming Wu, Tao Jiang, Xiaoyun Chen

**Affiliations:** 1 Department of Otolaryngology, the First Affiliated Hospital of Wenzhou Medical University, Wenzhou, Zhejiang, 325000 China; 2 Institute of Translation Medicine, the First Affiliated Hospital of Wenzhou Medical University, Wenzhou, Zhejiang, 325000 China

**Keywords:** Vocal cord leukoplakia, Long non-coding RNAs, Gene chip, Microarray

## Abstract

**Background:**

Long non-coding RNAs (lncRNAs) play an important role in tumorigenesis. Vocal cord leukoplakia is a precancerous lesion in otolaryngological practice. Till now, the expression patterns and functions of lncRNAs in vocal cord leukoplakia have not been well understood. In this study, we used microarrays to investigate the aberrantly expressed lncRNAs and mRNAs in vocal cord leukoplakia and adjacent non-neoplastic tissues.

**Methods:**

Gene Ontology and pathway analyses were performed to determine the significant function and pathways of the differentially expressed mRNAs. qRT-PCR was performed to further validate the expression of selected lncRNAs and mRNAs in vocal cord leukoplakia.

**Results:**

Our study identified 170 differentially expressed lncRNAs and 99 differentially expressed mRNAs, including 142 up-regulated lncRNAs and 28 down-regulated lncRNAs, and 54 up-regulated mRNAs and 45 down-regulated mRNAs. Among these, XLOC_000605 and DLX6-AS1 were the most aberrantly expressed lncRNAs. Furthermore, we identified an antisense lncRNA (LOC100506801), an enhancer-like lncRNA (AK057351) and three long intergenetic noncoding RNAs including XLOC_008001, XLOC_011989 and XLOC_007341.

**Conclusions:**

Our results revealed that many lncRNAs were differentially expressed between vocal cord leukoplakia tissues and normal tissue, suggesting that they may play a key role in vocal cord leukoplakia tumorigenesis.

## Background

Leukoplakia is a term to describe a mucosal white patch or plaque that cannot be easily scraped off. Vocal cord leukoplakia is a common precancerous lesion in otolaryngological practice. The annual incidence in the United States is estimated to be 10.2/100000 in males and 2.1/100000 in females. A comprehensive meta-analysis of laryngeal leukoplakia by Isenberg et al. revealed that 8.2% cases underwent malignant transformation during a follow-up period that ranged from 1 to 233 months between various studies. Overall 3.7% nondysplastic, 10.1% mild to moderate dysplastic and 18.1% severely dysplastic cases underwent malignant change [1]. Studies have identified smoking and alcohol as major causes and there is also sufficient evidence implicating gastroesophageal reflux and human papilloma virus in the pathogenesis of the disease [2].

Vocal cord leukoplakia is clinically significant due to the potential for malignant transformation. A variety of proliferation markers, cyclin kinases, oncoproteins, tumor suppressors, mutations microsatellite loss of heterozygosity (LOH), nuclear image parameters and DNA ploidy have been investigated in laryngeal dysplasias, which has provided insight into the molecular mechanism of carcinogenesis [3–5]. Bartlett et al. also identified several genes including IGF-1, EPDR1, MMP-2, S100A4 which were differentially expressed between vocal cord leukoplakia and normal vocal cord tissues [6]. Despite many investigations, the exact mechanism of vocal cord leukoplakia tumorigenesis remains unclear.

Recently, a new class of noncoding RNAs, designated long noncoding RNAs (lncRNAs), was found to be frequently dysregulated in various diseases. LncRNAs are transcript RNA molecules longer than 200 nucleotides that do not encode a protein and reside in the nucleus or cytoplasm [7]. Aberrant expression of lncRNAs can lead to abnormalities in gene expression and tumorigenesis. The altered expressions of lncRNAs are a feature of many types of cancers and have been shown to promote the development, invasion, and metastasis of tumors by a variety of mechanisms [8]. Studies have shown that lncRNAs play an important role in larynx squamous cell carcinoma (LSCC) progression. Shen et al. reported that AC026166.2–001 was the most down-regulated lncRNA and RP11-169D4.1–001 was the most up-regulated lncRNA in LSCC tissue compared to normal laryngeal tissue [9]. Some other lncRNAs also have been reported to be correlated with LSCC tumorigenesis and progression [10–14]. However, the role of lncRNAs in vocal cord leukoplakia tumorigenesis remains unclear.

In this study, we used gene microarray analysis to measure the expression patterns of lncRNAs and mRNAs in vocal cord leukoplakia samples and compared them with the corresponding patterns in adjacent nontumorous tissue (NT) samples. Several of the differentially expressed lncRNAs were evaluated by SYBR RT-PCR in 100 pairs of tissue samples. Our results suggest that the dysregulation of lncRNAs might play an important role in vocal cord leukoplakia tumorigenesis.

## Methods

### Patients samples

Vocal cord leukoplakia samples and control normal vocal cord mucosal samples were collected from 103 patients of the Department of Otolaryngology, First Affiliated Hospital of Wenzhou Medical University, China, from June 2015 to June 2016. Three samples were used for microarray analysis of lncRNAs and 100 were used for quantitative PCR (Q-PCR) validation. The clinical characteristics of patients with leukoplakia vs normal tissue (control) used in gene microarray were shown in Table 1. The diagnosis of vocal cord leukoplakia was based on clinical history and white light laryngoscopy findings and further confirmed by histopathologic diagnosis of parakeratosis and mild to severe dysplasia. The vocal cord leukoplakia and matched normal vocal cord mucosal samples were snap-frozen in liquid nitrogen immediately after resection. This study was approved by the Institutional Ethics Review Board of the First Affiliated Hospital of Wenzhou Medical University, and all patients provided written informed consent for this study.

**Table 1:** Clinical characteristics of patients with leukoplakia vs normal tissue used in gene microarray (*n* = 103)

	Age	Gender		Smoking	Alcohol Drinking	GERD
		Male	Female			
Normal	42.3 ± 5.7	97 (94.2%)	6 (5.8%)	45 (43.7%)	34 (33.0%)	6 (5.8%)
Leukoplakia	45.8 ± 6.9	99 (96.1%)	4 (4.9%)	87 (84.5%)	72 (69.9%)	37 (35.9%)

GERD: Gastroesophageal Reflux Disease

### RNA extraction

Vocal cord leukoplakia samples and normal vocal cord mucosal samples were obtained by biopsy under white light laryngoscopy. Total RNA was extracted using Trizol reagent (Invitrogen, Carlsbad, CA, USA), according to the manufacturer’s protocol. The integrity of the RNA was assessed by electrophoresis on a denaturing agarose gel. A NanoDrop ND-1000 spectrophotometer was used for the accurate measurement of RNA concentration (OD260), protein contamination (OD 260/OD 280 ratio), and organic compound contamination (OD 260/OD 230 ratio).

### Microarray and computational analysis

For microarray analysis, an Agilent Array platform (Agilent Technologies, Santa Clara, CA, USA) was employed. The microarray analysis was performed as described by our colleagues [15]. Briefly, sample preparation and microarray hybridization were performed based on the manufacturer’s standard protocols with minor modifications. Briefly, mRNA was purified from total RNA after removal of rRNA by using an mRNA-ONLY Eukaryotic mRNA Isolation Kit (Epicentre Biotechnologies, USA). Then, each sample was amplified and transcribed into fluorescent cRNA along the entire length of the transcripts without 3′ bias by using a random priming method. The labeled cRNAs were hybridized onto a Human lncRNA Array v3.0 (8 × 60 K; Arraystar), which was designed for 30,586 lncRNAs and 26,109 coding transcripts. The lncRNAs were carefully constructed using the most highly respected public transcriptome databases (RefSeq, UCSC Known Genes, GENCODE, etc.) as well as landmark publications. Each transcript was accurately identified by a specific exon or splice junction probe. Positive probes for housekeeping genes and negative probes were also printed onto the array for hybridization quality control. After washing the slides, the arrays were scanned using an Agilent G2505C scanner, and the acquired array images were analyzed with Agilent Feature Extraction software (version 11.0.1.1). Quantile normalization and subsequent data processing was performed using the GeneSpring GX v12.0 software package (Agilent Technologies). The microarray work was performed by KangChen Bio-tech, Shanghai, People’s Republic of China.

### Functional group analysis

We used Gene Ontology analysis (GO: http://www.geneontology.org) and pathway analysis to determine the function and pathways of the differentially expressed mRNAs in vocal cord leukoplakia tissues compared to adjacent control vocal cord tissues. The *P*-value denotes the significance of GO Term enrichment in the differentially expressed mRNA list (*P* < 0.05 was considered statistically significant). The pathway analyses for the differentially expressed mRNAs were performed based on the latest Kyoto Encyclopedia of Genes and Genomes (KEGG: http://www.genome.ad.jp/kegg/). This analysis allowed us to determine the biological pathways for which a significant enrichment of differentially expressed mRNAs existed (*P* < 0.05 was considered statistically significant).

### Quantitative PCR

Total RNA was extracted from frozen vocal cord leukoplakia tissues by using TRIzol reagent (Invitrogen) and then reverse-transcribed using an RT Reagent Kit (Thermo Scientific), according to the manufacturer’s instructions. LncRNAs expression in vocal cord leukoplakia tissues was measured by quantitative PCR by using SYBR Premix Ex Taq and an ABI 7000 instrument. Some candidate lncRNAs were validated by SYBRP PCR, these genes’ primers in the study for Q-PCR. Total RNA (2 mg) was transcribed to cDNA. PCR was performed in a total reaction volume of 20 μl, including 10 μl of SYBR Premix (2×), 2 μl of cDNA template, 1 μl of PCR forward primer (10 mM), 1 μl of PCR reverse primer (10 mM), and 6 μl of double-distilled water. The quantitative real-time PCR reaction included an initial denaturation step of 10 min at 95 °C; 40 cycles of 5 s at 95 °C, 30 s at 60 °C; and a final extension step of 5 min at 72 °C. All experiments were performed in triplicate, and all samples were normalized to GAPDH. The median in each triplicate was used to calculate relative lncRNAs concentrations (△Ct = Ct median lncRNA - Ct median GAPDH), and the fold changes in expression were calculated [16].

### Statistical methods

All results are represented as mean ± standard deviation. Statistical analysis was performed for the comparison of two groups in the microarray, and analysis of variance for multiple comparisons was performed the Student’s t-test using SPSS software (Version 17.0 SPSS Inc.). A value of *p* < 0.05 was considered statistically significant.

The fold change and the Student’s t-test were used to analyze the statistical significance of the microarray results. The false discovery rate (FDR) was calculated to correct the *P*-value. The threshold value used to designate differentially expressed lncRNAs and mRNAs was a fold change ≥2.0 or ≤0.5 (*P* < 0.05).

## Results

### Overview of lncRNA profiles

**Fig. 1:**
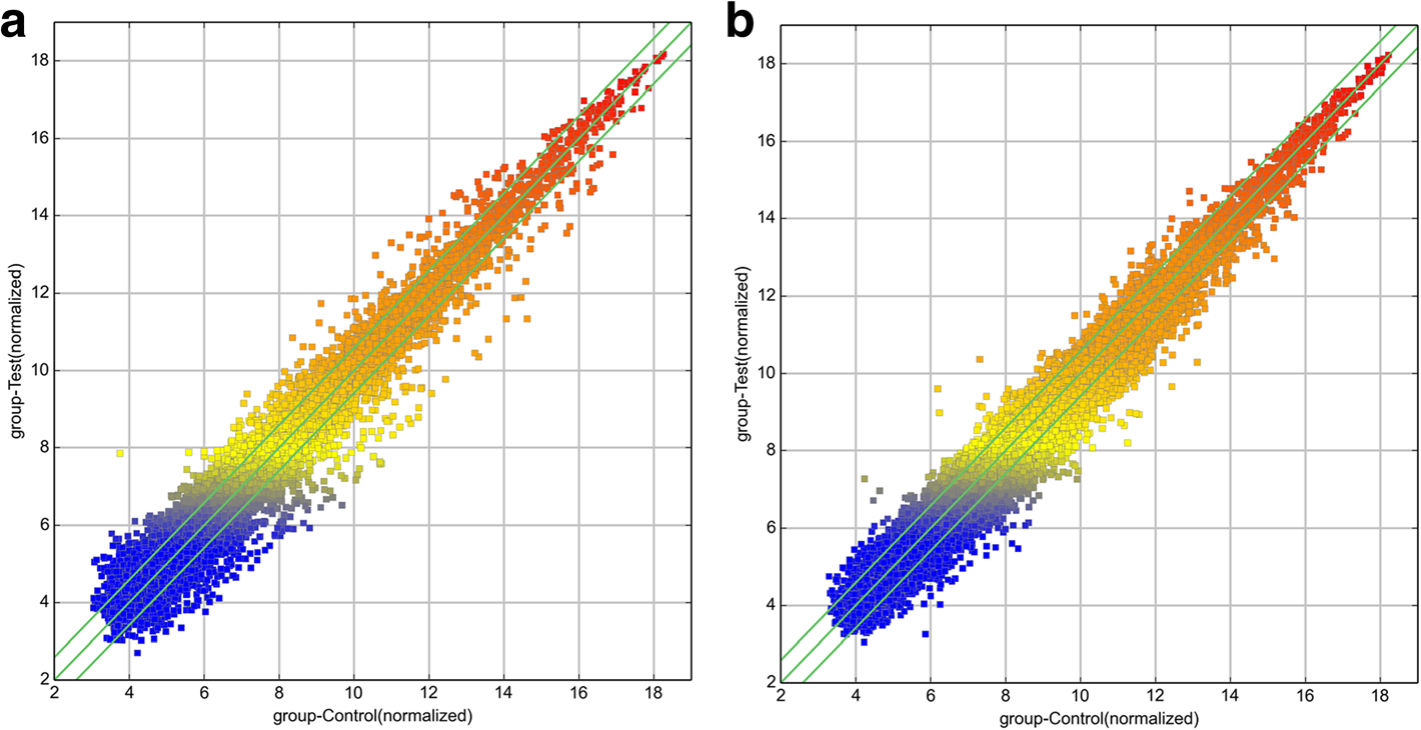
**a**–**b** Scatter plots showing the variation in lncRNA (**a**) and mRNA (**b**) expression between the vocal cord leukoplakia and normal vocal cord tissue arrays. The values of the X and Y axes in the scatter plot are averaged normalized values in each group (log2-scaled). The lncRNAs above the top green line and below the bottom green line are those with a > 3-fold change in expression between the two tissues

**Fig. 2:**
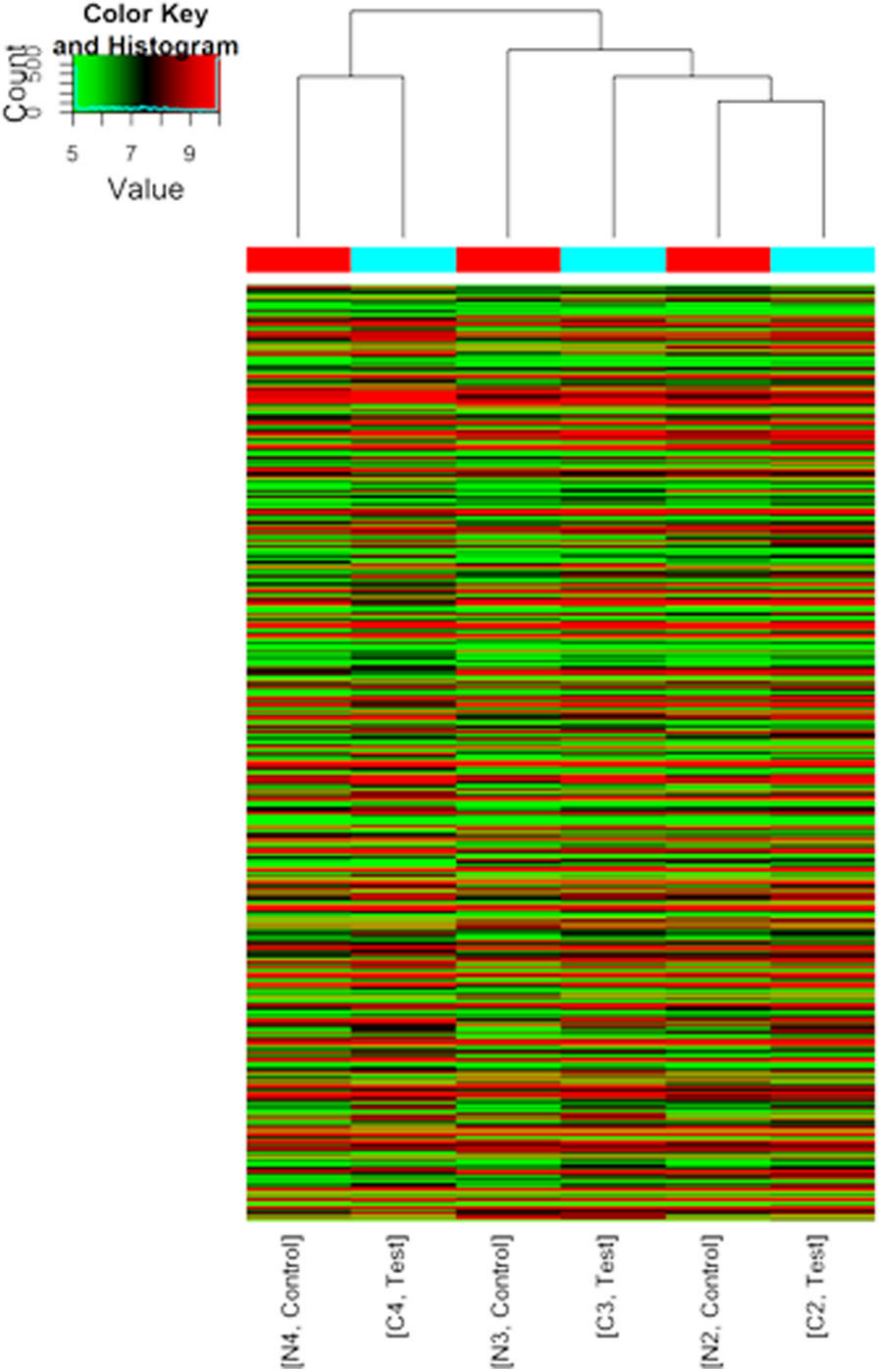
Heat map and hierarchical clustering of lncRNA profile comparison between the vocal cord leukoplakia and normal vocal cord samples. Red color indicates over expression and green color indicates low expression. Every column represents a tissue sample and every row represents an lncRNA probe. C represents leukoplakia tissues and N represents adjacent normal tissues

To study the potential biological functions of lncRNAs in vocal cord leukoplakia, we examined the lncRNA and mRNA expression profiles in human leukoplakia by microarray analysis (Figs. 1 and 2). In this study, authoritative data sources containing more than 30,586 lncRNAs were used to study the potential biological functions of lncRNA and mRNA expression profiles in vocal cord leukoplakia through microarray analysis. Our results showed that there were 170 differentially expressed lncRNAs (fold change ≥2.0 or ≤0.5; *P* < 0.05) between vocal cord leukoplakia and normal vocal cord samples. Among these, 142 lncRNAs were found to be up-regulated in the vocal cord leukoplakia group compared to the normal vocal cord mucosal group, while 28 lncRNAs were down-regulated between these two groups (Table 2 shows the top 10 differentially expressed lncRNAs). Among these, XLOC_000605 was the most significantly up-regulated lncRNA and DLX6-AS1 was the most significantly down-regulated one.

**Table 2:** Top 10 differentially expressed lncRNAs in vocal cord leukoplakia tissue compared with adjacent non-tumorous tissue

up-regulated		down-regulated	
lncRNAs	Fold Change	lncRNAs	Fold Change
XLOC_000605	17.24	DLX6-AS1	4.14
RP11-187O7.3	6.17	KRT17P2	4.13
XLOC_011401	5.31	RP13-608F4.1	3.08
SACS-AS1	4.98	L25629	2.90
XLOC_011403	4.92	CTD-2382E5.1	2.63
FAM86FP	4.11	RP11-351E7.1	2.58
LOC100131138	3.96	HERC2P2	2.49
AC005152.2	3.86	SAA3P	2.48
AC004920.3	3.82	XLOC_006684	2.43
XLOC_008001	3.78	VNN2	2.36

### LncRNAs classification and subgroup analysis

#### Differentially expressed antisense lncRNAs and nearby coding genes

Mammalian genomes encode numerous natural antisense transcripts. Functional validation studies indicate that antisense transcripts are not a uniform group of regulatory RNAs but instead belong to multiple categories with some common features. Recent evidence indicates that antisense transcripts are frequently functional and use diverse transcriptional and post-transcriptional gene regulatory mechanisms to carry out a wide variety of biological roles [17]. In this study, LOC100506801 was the only differentially expressed antisense lncRNA (fold change ≥2.0, *P* < 0.05) between vocal cord leukoplakia and normal vocal cord samples. It was significantly up-regulated as was its nearby gene, ECE19 (fold change = 1.70, *P* = 0.001).

**Fig. 3:**
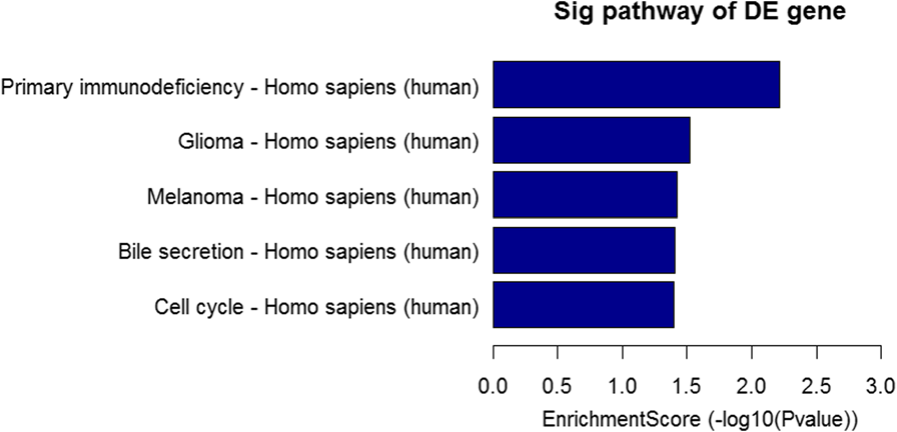
Pathway analysis of upregulated mRNAs in vocal cord leukoplakia. Five upregulated pathways were identified, including Primary immunodeficiency, Glioma, Melanoma, Bile secretion, Cell cycle signaling pathways

#### Differentially expressed enhancer-like lncRNAs and nearby coding genes

Ørom UA et al. found an enhancer-like function for a set of lncRNAs in human cell lines. Depletion of these lncRNAs led to decreased expression of their neighboring protein-coding genes [18]. In this study, we identified the lncRNAs with enhancer-like lncRNA functions using GENCODE annotation. Our results reveal that AK057351 was the only differentially expressed enhancer-like lncRNA (fold change ≥2.0, *P* < 0.05) between these two groups. It was up-regulated and its nearby gene was EFHA1. EFHA1 was itself up-regulated like the enhancer-like lncRNA (fold change =2.43, *P* = 0.03).

#### Differentially expressed lincRNAs and associated coding gene

Long intergenetic noncoding RNAs (lincRNAs) are transcribed from thousands of loci in mammalian genomes and might play widespread roles in gene regulation and other cellular processes [19]. In this study, we identified 3 differentially expressed lincRNAs and associated coding mRNAs (fold change ≥2.0, *P* < 0.05): XLOC_008001, XLOC_011989 and XLOC_007341. All of them were up-regulated as were their associated mRNAs, MSN (fold change =1.63, *P* = 0.01), RRAD (fold change =2.69, *P* = 0.04) and TPM2 (fold change =1.68, *P* = 0.007), respectively.

### Overview of mRNA profiles

Ninety-nine mRNAs were found to be differentially expressed between vocal cord leukoplakia and normal vocal cord mucosa tissue (fold change ≥2.0, *P* < 0.05). Among these, 54 were up-regulated and 45 were down-regulated (Table 3 shows the top 10 differentially expressed mRNAs).

**Table 3:** Top 10 differentially expressed mRNAs in vocal cord leukoplakia tissue compared with adjacent non-tumorous tissue

up-regulated		down-regulated	
mRNAs	Fold Change	mRNAs	Fold Change
RPL10L	3.79	GPX8	3.99
SOWAHA	3.51	WDR19	3.87
HMGCS2	3.38	SEC31A	3.45
ZSCAN1	3.17	CTSF	3.42
OR4P4	2.97	ARID4A	3.14
PDP1	2.93	KIF20A	2.99
C1orf53	2.82	DUSP6	2.89
OSGIN2	2.81	CALD1	2.86
ZNF853	2.79	PNISR	2.82
OR6C3	2.78	FIP1L1	2.81

### GO analysis

GO analysis is a functional analysis that associates differentially expressed mRNAs. The GO categories were derived from the Gene Ontology website (www.geneontology.org) and comprised of 3 structured networks: biological processes, cellular components and molecular function. According to the GO annotation tool, the genes corresponding to the down-regulated mRNAs included 455 genes involved in biological processes, 73 genes involved in cellular components and 60 genes involved in molecular functions. The genes corresponding to the up-regulated mRNAs included 109 genes involved in biological processes, 12 genes involved in cellular components, and 21 genes involved in molecular functions.

### Pathway analysis

**Fig. 4:**
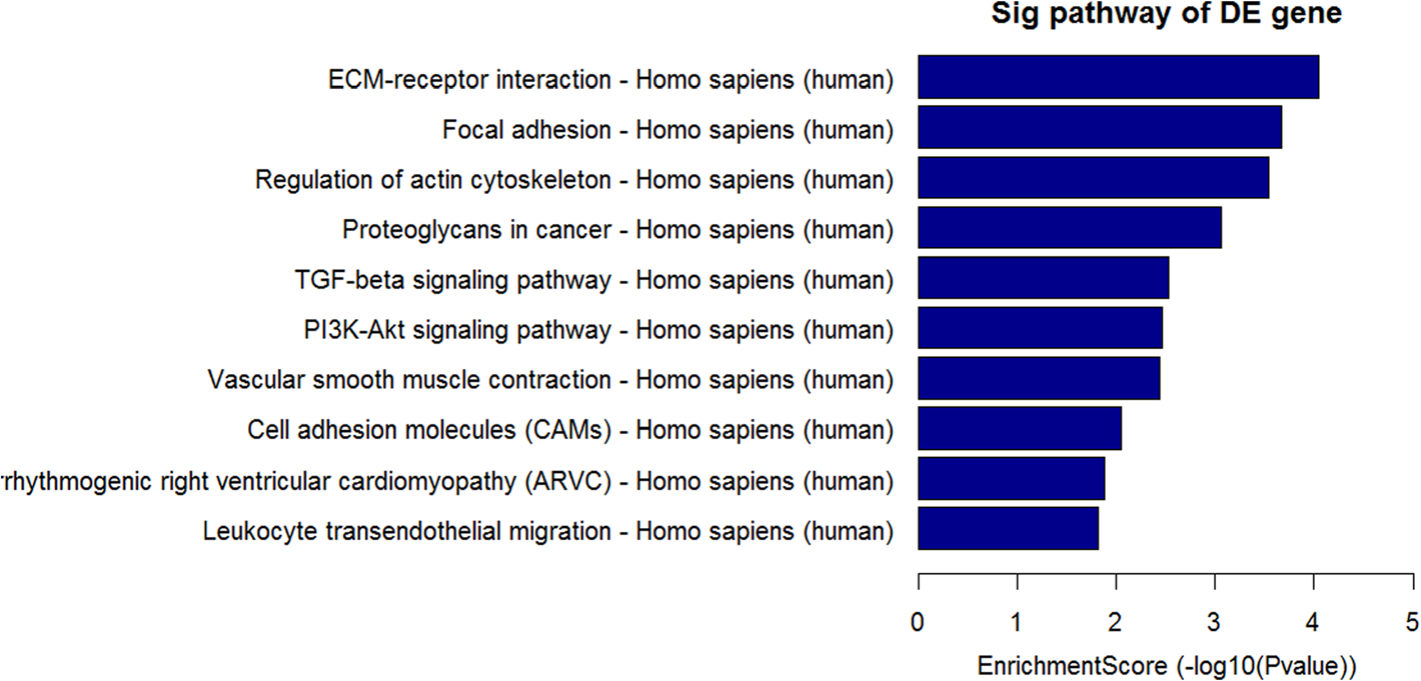
Pathway analysis of downregulated mRNAs in vocal cord leukoplakia. Fifteen downregulated pathways were identified, including ECM-receptor interaction, focal adhesion, Regulation of actin cytoskeleton, Proteoglycans in cancer, TGF-beta signaling pathway, Cell adhesion molecules and PI3K-Akt signaling pathways

We performed the pathway analysis based on the latest Kyoto Encyclopedia of Genes and Genomes (KEGG) database. This analysis was used to determine the biological pathways associated with the most differentially expressed mRNAs in vocal cord leukoplakia. Our results identified 5 up-regulated pathways (including Primary immunodeficiency, Glioma, Melanoma, Bile secretion, Cell cycle signaling pathways) (Fig. 3) and 14 down-regulated pathways (including ECM-receptor interaction, focal adhesion, Regulation of actin cytoskeleton, Proteoglycans in cancer, TGF-beta signaling pathway, Cell adhesion molecules and PI3K-Akt signaling pathways) (Fig. 4).

### Real-time quantitative PCR validation

**Fig. 5:**
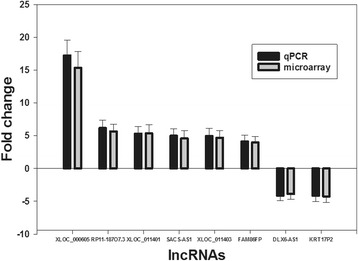
Comparison between gene chip data and qPCR result. XLOC_000605, RP11-187O7.3, XLOC_011403, XLOC-011401, SACS-AS1, FAM86FP, DLX6-AS1, KRT17P2 determined to be differentially expressed in vocal cord leukoplakia samples compared with NT samples in three patients by microarray were validated by qPCR. The heights of the columns in the chart represent the log-transformed median fold changes (T/N) in expression across the three patients for each of the four lncRNAs validated. The validation results of the 8 lncRNAs indicated that the microarray data correlated well with the qPCR results

Based on features of the differentially expressed lncRNAs such as fold difference, gene locus, and nearby encoding genes, a number of interesting candidate lncRNAs were selected for further analysis (including XLOC_000605, RP11-187O7.3, XLOC_011403, XLOC-011401, SACS-AS1, FAM86FP, DLX6-AS1, KRT17P2). We verified the expression of these lncRNAs by real-time quantitative RT-PCR by using GAPDH as a reference gene and by calculating the 2^-△△CT^ values. The results showed that the microarray results for the selected lncRNAs were consistent with the results of RT-PCR (Fig. 5).

## Discussion

In recent years, researchers have focused their attention on the analysis of protein-coding transcripts to characterize patterns and potential functional roles. The development of next-generation sequencing technology has led to the discovery of a new class of non-coding RNA transcripts, lncRNAs. Numerous investigations suggest that lncRNAs perform key regulatory functions in chromatin remodeling and gene expression in many biological processes, including X-chromosome inactivation, gene imprinting, and stem cell maintenance [20, 21]. Furthermore, lncRNAs are important factors in the control of gene expression in cancer [22], and lncRNAs such as HOTAIR have been shown to play a significant role in the development and progression of tumors [8]. It has also been demonstrated that lncRNAs are differentially expressed in normal cells and tumor cells. As lncRNAs constitute an important class of gene expression regulatory factors, their aberrant expression would inevitably lead to abnormal gene expression levels, which may result in tumorigenesis. Promoters bind to many transcription factors by mechanisms such as chromosomal rearrangements and transfer elements [23]. However, the profile and the biological function of lncRNAs in vocal cord leukoplakia remain unknown.

Until now, there have been no reports describing the expression profiles of lncRNAs in vocal cord leukoplakia and there have been no studies on the association of lncRNA expression with the clinical characteristics and outcomes of in vocal cord leukoplakia. In this study, we analyzed the lncRNAs expression profiles in the tissues of vocal cord leukoplakia to uncover the potential role of lncRNAs in the pathogenesis of its tumorigenesis. High-throughput microarray techniques revealed a set of differentially expressed lncRNAs, including 142 that were up-regulated and 28 that were down-regulated in vocal cord leukoplakia tissue compared to normal vocal cord mucosa. Furthermore, we identified several subgroups of lncRNA, including antisense lncRNA, enhancer-like lncRNA, and lincRNA. Enhancers are classically defined as *cis*-acting DNA sequences that can increase the transcription of genes. They generally function independently of orientation and at various distances from their target promoter (or promoters) [24]. Ørom et al. also found some lncRNAs with enhancer-like functions in human cells [18]. In this study, we identified a significantly up-regulated enhancer-like lncRNA AK057351 and its associated gene EFHA1. Antisense lncRNAs are another subgroup of lncRNAs which can induce chromatin and DNA epigenetic changes, thus affecting the expression of sense mRNA. In this study, we identified an up-regulated antisense lncRNA LOC100506801 and its associated gene, ECE19. LincRNA are long non-coding sequences located between the protein-coding genes. More than 3500 lincRNAs have been reported in mammalian genome so far, which are involved in physiological processes through regulation of gene expression. Aberrant expression of lincRNAs has been found in both solid tumors and leukemia. The role of lincRNAs, however, remains unclear. In this study, we identified 3 significantly up-regulated lincRNAs and associated coding mRNAs. They were XLOC_008001, XLOC_011989 and XLOC_007341 and the associated mRNAs were MSN, RRAD and TPM2, respectively.

To investigate the lncRNAs’ target gene function, GO analysis and KEGG pathway annotation were applied to the lncRNAs’ target gene pool. GO analysis revealed that the number of genes corresponding to down-regulated mRNAs was larger than that corresponding to up-regulated mRNAs. KEGG annotation showed that there were 5 up-regulated pathways (including ethanol metabolism, viral carcinogenesis, RNA transduction, and cell cycle pathways) and 14 down-regulated pathways (including propionate metabolism and fatty acid metabolism pathways). These pathways might play important roles in vocal cord leukoplakia tumorigenesis. Further studies should be performed to investigate this hypothesis. 8 of the lncRNAs identified in the microarray analysis were confirmed by RT-PCR to be aberrantly expressed in vocal cord leukoplakia tissues. Among these lncRNAs, XLOC_000605 was the most significantly up-regulated, and DLX6-AS1 was the most significantly down-regulated. Little has been known about the function of these two lncRNAs until now. These findings may provide a potential strategy to distinguish between vocal cord leukoplakia tissue and normal vocal cord tissue. Our results suggest that these two lncRNAs might contribute to vocal cord leukoplakia tumorigenesis. Further studies of the biological function of XLOC_000605 and DLX6-AS1 will be required to confirm this potential association.

## Conclusions

In conclusion, our study revealed a set of lncRNAs with differential expression in vocal cord leukoplakia compared with normal larynx mucous tissue, and also identified several subgroups of lncRNAs such as antisense lncRNAs, enhancer-like lncRNAs and lincRNAs. Moreover, we found that XLOC_000605 and DLX6-AS1 were significantly dysregulated and these two lncRNAs might contribute to vocal cord leukoplakia tumorigenesis. One limitation to this study is the small sample size, which may have been insufficient to detect every truly differentially expressed gene. In addition, we did not investigate the function of the differentially expressed genes which were identified. Further investigations directed at the lncRNAs and mRNAs identified above will be required to uncover their biological functions and their association with vocal cord leukoplakia tumorigenesis.

## Abbreviations

GO: Gene Ontology
KEGG: Kyoto Encyclopedia of Genes and Genomes
lincRNA: long intergenetic noncoding RNA
lncRNA: long non-coding RNA
NT: nontumorous tissue
